# Alcohol abstinence, non-hazardous use and hazardous use a decade after alcohol-related hospitalization: registry data linked to population-based representative postal surveys

**DOI:** 10.1186/1471-2458-14-874

**Published:** 2014-08-24

**Authors:** Kozma Ahacic, Robert F Kennison, Ingemar Kåreholt

**Affiliations:** Department of Public Health Sciences, Karolinska Institutet, Tomtebodavägen 18A, 171 77 Stockholm, Sweden; Centre for Epidemiology and Community Medicine, Health Care Services, Stockholm County Council, Box 1497, 171 29 Solna, Sweden; Department of Psychology, California State University, Los Angeles, California; Aging Research Centre (ARC), Karolinska Institutet and Stockholm University, Gävlegatan 16, 113 30 Stockholm, Sweden; Institute of Gerontology, School of Health Sciences, Jönköping University, Jönköping, Sweden

**Keywords:** Treatment, Rehospitalization, Alcohol use disorder (AUD), Controlled drinking, Alcohol dependence, Harmful use of alcohol

## Abstract

**Background:**

Although there is evident association between alcohol-related hospitalization and alcohol use, the relationship has not been well examined. This study analyzed the extent of alcohol abstinence, non-hazardous use and hazardous use among people who had experienced alcohol-related hospitalization during the preceding decade.

**Method:**

Registry data concerning alcohol-related hospitalizations between 1996 and 2007 were linked to two representative surveys, in 2006 and 2007, of residents of Stockholm County. Relevant contrasts were modeled, using logistic regression, in the pooled sample (n = 54 955). Ages were 23–84 years at follow-up.

**Results:**

Among persons previously hospitalized (n = 576), half reported non-hazardous use. Non-hazardous use was less prevalent than in the general population – and the extent of non-hazardous use did not change over time following hospitalization. There were no significant age differences, but non-hazardous use was less frequent among people with repeated episodes of care. One in six was abstinent. Abstinence was more common among the old, while hazardous use (exceeding 14 drinks per week for men, and 9 drinks per week for women) decreased with age. Abstinence also increased over time; among persons hospitalized ten years ago, the abstinence rate was twice that of the general population. Associations with hazardous use over time were less conclusive. Hazardous use among those previously hospitalized decreased over time in one sample but not in the other. After pooling the data, there were indications of a decrease over time following hospitalization, but more prevalent hazardous use than in the general population.

**Conclusions:**

Following alcohol-related hospitalization, abstinence increased, and there was no evidence of regression towards the mean, i.e., towards non-hazardous use. Abstinence was also more widespread among previously hospitalized persons of older ages. With advancing age, changing hazardous alcohol habits among previously hospitalized appears to yield a trend towards promotion of abstinence.

## Background

Excessive use of alcohol is one of the leading causes of morbidity and mortality worldwide [1]. It is the fifth most important modifiable health behavior, having consequences not only for the individual but also for family and friends. Society incurs a great cost from the behavioral and medical effects of alcohol use, amounting to several percent of gross domestic product [2].

Approximately 15% of the Swedish population has been estimated to exhibit hazardous alcohol use, where hazardous use is defined as an average consumption exceeding 14 drinks per week for men, and 9 drinks per week for women [3]. Urban areas, such as Stockholm, have been associated with slightly higher estimates of alcohol consumption [4]. And it has been estimated that about one percent of members of the Swedish population have more severe alcohol use disorders, i.e., indications of harmful use and alcohol dependence. These disorders are associated with numerous behavioral, cognitive, and physiological symptoms.

In order to assess the potentially negative consequences of alcohol use, yearly rates of alcohol-related hospitalizations are monitored, both in Sweden and elsewhere. During an average year, approximately one percent of Stockholm’s population is hospitalized with an alcohol-related diagnosis [5]. People who have experienced alcohol-related hospitalization represent an at risk group that includes the most severe cases of alcohol use disorders.

Among those previously hospitalized, there is a high degree of recidivism, i.e., return to alcohol-related care, which is likely to be associated with continuing excessive alcohol use [6]. An analysis of follow-up data indicates that, over a ten-year period, approximately two thirds of people hospitalized for alcohol-related issues returned for further inpatient care, while one third never returned [6].

Of those who received alcohol-related inpatient care in Stockholm in 1997, 62 percent were identified as being alcohol dependent, and 18 percent as harmful users [6]. Yet, it should be noted that there is no medical diagnosis that specifically identifies hazardous use as a distinct category separate from harmful use or alcohol dependence. Also, it is possible for a single episode of excessive alcohol use to result in hospitalization. For example, minors may be forcibly admitted to hospital by police for detoxification and observation. A large portion of those who are hospitalized for inpatient care are recidivists, which is why the identification of harmful use and dependence predict recurrent care episodes [6].

By studying the alcohol-related behavior of people with previous alcohol-related hospitalization, it is possible to derive measures of the efficacy of treatment. Such information may be useful for the creation of best practices related to interventions for those in need of care. Currently, for hazardous users, education and brief counseling are the recommended interventions; for harmful use, education and counseling with follow-up to monitor future behavior are recommended [7, 8]; and, for alcohol dependency, more advanced treatments, such as detoxification and inpatient care, are recommended. All inpatients are offered alcohol treatment, but the extent to which these patients follow through with their prescribed treatment remains unclear. While some alcohol users require elaborate interventions from health care providers, most heavy users recover naturally without formal treatment [9–13]. For people who have alcohol behavior issues, a hospitalization may represent “a teachable moment.”

There is a clear need to study alcohol habits among those with prior hospitalizations, and this is the first study to provide prevalence estimates for this population. Other issues that remain unanswered are whether there are gender and age differences in the prevalence of non-hazardous use and abstinence following hospitalization [6, 14], and whether alcohol consumption habits change with the passage of time following hospitalization.

This study aims to report on the extent of alcohol abstinence, non-hazardous use, and hazardous use among people who experienced alcohol-related hospitalization during the preceding ten years of their lives.

## Methods

Stockholm County had 1.4 million inhabitants in 1997, which was the baseline year for the study, and is an expanding metropolitan region. During 1997 the County registered approximately 5,400 patients with at least one alcohol-related inpatient care episode [6]. The public health care programs offered at the time included detoxification and specialized treatment. The County provides two specialized emergency units for addictive diseases to care for patients with acute substance-related conditions. Although some patients may be the responsibility of other hospitals and emergency departments, most patients with an urgent alcohol-related need for medical or psychiatric attention are served by or transferred to these specialized units.

One of two 24-hour outpatient units is likely to provide alcohol-related treatment for people suffering from intoxication in the Greater Stockholm area. These patients may be transferred from urgent care following medical clearance, self-admission, or being brought in by police. Patients who require close supervision, or more than 6 hours to achieve sobriety, are transferred to an inpatient unit. Patients with a high risk of neuropsychological conditions, such as withdrawal seizure or alcohol-withdrawal delirium, may also be transferred to a designated inpatient unit for medical care and observation.

Admission records and treatment data were obtained from the Stockholm County Inpatient Care Register for the time period 1997 to 2007. These registry data are considered reliable, and have been previously used to track time trends in alcoholic disorders [15–17].

### The sample

The study sample was based on the combined data from two mail-in surveys carried out in 2006 and 2007; they were each representative of the population of Stockholm County (n = 58 506, response rate 55.3 percent). The 2006 survey (n = 34 707, response rate 61.3 percent) was a cross-sectional study, while the 2007 survey (n = 23 797, response rate 49.7 percent) was a follow-up to a longitudinal study initiated in 2002. The respondents from the baseline testing in 2002 were re-recruited in 2007, excluding those who had moved or died. The baseline response rate was 62.5 percent in 2002, while the follow-up response rate was 79.6 percent in 2007. The 2006 survey included persons born between 1922 and 1988, while the 2007 survey included those born between 1918 and 1984. To get the same age span at baseline, i.e., 13–74 years, the 1921–1983 birth cohorts (n = 32 626) were included from the 2006 survey, and the 1922–1984 birth cohorts (n = 23369) from the 2007 survey. From among these respondents, three percent (n = 1735) were excluded from the analysis because they failed to respond to one or more of the items concerning alcohol use, which was necessary for categorization of the outcome variables.

In responding to the surveys, respondents consented to have their survey data linked to health registers. The surveys were administered by Statistics Sweden. Linking the survey responses to national registers gave information about the respondents’ alcohol-related hospitalizations. The study was approved by the local ethical committee in Stockholm of the Central Ethical Review Board (diary number: 2010/704).

### Measures

#### Alcohol consumption

Alcohol consumption during a typical week was measured using a beverage-specific grid from the 2006 survey. It is a period-specific normal week (PSNW) measure [18, 19]. The grid has four rows, with a single row comprising the first four days of the week, and with separate rows for the weekend days, Friday, Saturday, and Sunday. Different columns correspond to the types of beverages: spirits, fermented wine, wine, beer, and low-alcohol beer. The responders filled in the estimated volume they had consumed by day of the week and beverage. In 2007, the AUDIT-C instrument [20, 21] was used instead of the grid. Both measures identified abstainers, hazardous alcohol users, and non-hazardous users. Hazardous use in the PNSW is defined as alcohol consumption in excess of 14 (men), or of 9 (women), normal glasses (equivalent to 12 g of pure alcohol) per week (which corresponds to scores of 8+ for men and 6+ for women in AUDIT-C) [22]. Consumption less than that was defined as non-hazardous.

#### Alcohol-related hospitalizations

The diagnoses used in the register follow the International Classification of Diseases (ICD-10) [23]. All care episodes in 1997–2006 and in 1998–2007, respectively, were examined for the following alcohol-induced or -related diagnoses: alcohol intoxication, corresponding to F10.0; acute intoxication due to alcohol, or T51 toxic effect of alcohol; harmful use of alcohol F10.1; alcohol dependence F10.2; alcohol-induced chronic pancreatitis K86.0; alcoholic liver disease K70; alcohol-induced pseudo-Cushing's syndrome E24.4; degeneration of nervous system due to alcohol G31.2; alcoholic polyneuropathy G62.1; alcoholic myopathy G72.1; alcoholic cardiomyopathy I42.6; alcoholic gastritis K29.2; maternal care for (suspected) damage to fetus from alcohol O35.4; fetus and new-born affected by maternal use of alcohol P04.3; fetal alcohol syndrome Q86.0; blood alcohol level Y90; alcohol intoxication Y91; alcohol rehabilitation Z50.2; alcohol abuse counseling and surveillance Z71.4; mental and behavioral disorders due to use of alcohol (F10), including withdrawal state F10.3, delirium F10.4, psychotic disorder F10.5 & F10.7, amnesic syndrome F10.6, and other mental and behavioural disorders F10.8 & F10.9.

Four different variables were created to capture hospitalization history: a dichotomous outcome that referred to whether people had been hospitalized with any of the above diagnoses or not; the number of years since last hospitalization; the number of hospitalization episodes; and, the number of years with hospitalization episodes. The last two, the number of episodes and the number of years with episodes, were needed to control for the second variable, the number of years since last hospitalization, in order to enable more reasonable comparisons within the models. These variables were assumed to be continuous in the reported regression models, but are presented as grouped measures in Tables 1 and 2.

**Table 1:** **Descriptive data**

	All		With previous alcohol-related hospitalizations	
	Percent	*n*	Percent	*n*
**Alcohol use**				
Abstinent	11.1	*6087*	16.3	*94*
Non-hazardous use	73.3	*40260*	51.6	*297*
Hazardous use	15.7	*8608*	32.1	*185*
	100.0	*54955*	100.0	*576*
**Previous hospitalizations**	1.3	*576*	100.0	*576*
No hospitalizations	98.7	*54379*	0.0	*0*
**Survey**				
2006	58.8	*32331*	64.6	*372*
2002/2007	41.2	*22624*	35.4	*204*
**Gender**				
Men	44.6	*24534*	62.0	*357*
Women	55.4	*30421*	38.0	*219*
**Age group**				
23-34	17.6	*9672*	12.5	*72*
35-44	21.1	*11549*	12.9	*74*
45-54	18.4	*10114*	20.2	*116*
55-64	20.9	*11453*	30.7	*176*
65-84	22.0	*12035*	23.7	*136*
**Years since last hospitalization**				
**Last hospitalized:**	99.0	*54379*	-	*0*
The same year	0.2	*125*	21.7	*125*
2-4 years ago	0.4	*210*	36.5	*210*
5-7 years ago	0.2	*137*	23.8	*137*
8-10 years ago	0.2	*104*	18.1	*104*
**Number of years with hospitalizations**				
0	99.0	*54379*	-	*0*
1	0.7	*370*	64.2	*370*
2	0.2	*100*	17.4	*100*
3+	0.2	*106*	18.4	*106*
**Number of hospitalizations**				
0	99.0	*54379*	-	*0*
1	0.5	*276*	47.9	*276*
2	0.2	*104*	18.1	*104*
3+	0.4	*196*	34.0	*196*

**Table 2:** **Percentage of abstainers, non-hazardous, and hazardous alcohol users by the subcategories of the independent variables for the entire sample and among the persons with previous alcohol-related hospitalizations**

	All			With previous alcohol- related hospitalizations		
	Abstinence	Non-hazardous use	Hazardous use	Abstinence	Non-hazardous use	Hazardous use
**Survey**						
2006	12.4	66.9	20.7	16.4	49.7	33.9
2002/2007	9.2	82.3	8.5	16.2	54.9	28.9
**Gender**						
Men	8.0	74.8	17.1	15.1	53.8	31.1
Women	13.5	72.0	14.5	18.3	48.0	33.8
**Age group**						
23-34	10.9	71.6	17.5	4.2	51.4	44.4
35-44	9.8	75.5	13.8	23.0	41.9	35.1
45-54	9.2	72.3	18.5	17.2	44.0	38.8
55-64	8.8	73.3	17.9	18.2	51.1	30.7
65-84	16.0	72.3	11.7	16.2	64.0	19.8
**No hospitalization**						
**Last hospitalized:**	11.0	73.5	15.5			
The same year	8.0	53.6	38.4			
2-4 years ago	15.7	49.0	35.2			
5-7 years ago	20.4	51.1	28.5			
8-10 years ago	22.1	54.8	23.1			
**Number of years with hospitalizations**						
0	11.0	73.5	15.5			
1	14.6	52.4	33.0			
2	19.0	57.0	24.0			
3+	19.8	43.4	36.8			
**Number of hospitalizations**						
0	11.0	73.5	15.5			
1	13.4	52.5	34.1			
2	15.4	53.8	30.8			
3+	20.9	49.0	30.1			

#### Analysis

First, bivariate relationships were estimated in the entire sample for the variables: hospitalization, survey, gender, and age group. Then, bivariate relationships were estimated separately among the hospitalized for the variables: survey, gender, age group, number of years since hospitalization, number of hospitalizations during a year, and number of years with hospitalization.

The first regression model included the independent variables for hospitalization, survey, gender, and age group for the entire sample, while the second model considered the same variables for the previously hospitalized. As well as survey, gender, and age group, the second model for the previously hospitalized included: number of years since last hospitalization, number of hospitalizations during a year, and number of years with hospitalization.

In a third model, all the estimated variable categories from the first two models were included. The contrasts for the variable categories among those with previous hospitalization were changed and set to show relevant interaction terms, i.e., whether the odds ratios between the variable categories among the hospitalized differed significantly from the corresponding odds ratios among the non-hospitalized.

Finally, to find possible deviations between survey patterns, the third model was estimated separately for the two surveys. If needed, suitable interaction terms for these possible deviations were then modeled. This was the case for the outcome, hazardous use, where results differed significantly between the two survey samples on the variable that measured change in odds over time since hospitalization.

The models were fitted to each of the three outcome measures: 1) abstinence in comparison with non-hazardous use or hazardous use, 2) hazardous use in comparison with non-hazardous use or abstinence, and 3) non-hazardous use in comparison with abstinence or hazardous use.

Logistic regression models using Proc Logistic in SAS 9.2 [24] were used to assess the relationships.

## Results

Just over one percent of the population was hospitalized during the ten-year follow-up period. Table 1 shows the prevalence of abstainers, non-hazardous users, and hazardous alcohol users combined, and of persons with previous alcohol-related hospitalizations separately. For the entire sample, 11% were abstainers, while 16% were abstainers among those with previous hospitalization. The corresponding figures for non-hazardous use were 73% and 52%; and, for hazardous use, 16% and 32%.

Among the previously hospitalized, respondents from the 2006 survey were overrepresented in comparison with the 2007 survey. Similarly, there were fewer persons in the younger age groups 23–34 and 35–44, and more in the 55–64 age group among the previously hospitalized in comparison with the general population. Men were overrepresented in comparison with women.

A less frequent outcome was having recently been hospitalized. Among those previously hospitalized, one fifth had been hospitalized during the preceding year, two fifths had been hospitalized during the three years before that, and two fifths had been hospitalized more than four years ago (including one fifth who had been hospitalized between seven and ten years ago). Half had been hospitalized more than once during follow-up. A third had been hospitalized during two or more separate years.

Table 2 shows the distribution of percentages of alcohol use in the different categories of the independent variables. The bivariate analyses shown in Tables 3, 4 and 5 give the corresponding relative probabilities and significance levels.

**Table 3:** **Odds ratios (OR) for abstaining from alcohol from logistic regression models with previous alcohol-related hospitalization, gender, age group, and interactions between hospitalization and; gender, age group, numbers of years after hospitalization, number of hospitalizations during a year, and number of years with hospitalizations as independent variables**

	Bivariate ^1^		Model 1 ^2^		Model 2		Model 3	
	OR	95 CI	OR	95 CI	OR	95 CI	OR	95 CI
**All**								
Non-hospitalized	1.0		1.0				1.0	
Hospitalized	1.58^***^	1.26-1.96	1.75^***^	1.39-2.19			0.11^***^	0.03-0.73
2006	1.0		1.0				1.0	
2002/2007 survey	0.72^***^	0.68-0.76	0.69^***^	0.65-0.73			0.69^***^	0.65-0.73
Men	1.0		1.0				1.0	
Women	1.79^***^	1.69-1.90	1.84^***^	1.74-1.95			1.85^***^	1.74-1.96
Age group								
23-34	1.0		1.0				1.0	
35-44	0.89^***^	0.81-0.97	0.92	0.84-1.01			0.90^*^	0.83-0.99
45-54	0.83^***^	0.76-0.91	0.87^**^	0.79-0.95			0.86^**^	0.78-0.94
55-64	0.80^***^	0.73-0.87	0.84^***^	0.77-0.92			0.83^***^	0.75-0.91
65-84	1.57^***^	1.45-1.70	1.69^***^	1.56-1.83			1.68^***^	1.55-1.82
**With previous alcohol-related hospitalizations**								
2006	1.0				1.0		1.0^3^	
2002/2007 survey	0.98	0.62-1.56			0.94	0.58-1.52	1.37	0.84-2.22
Men	1.0				1.0		1.0^4^	
Women	1.25	0.80-1.96			1.41	0.88-2.27	0.76	0.47-1.23
Age group								
23-34	1.0				1.0		1.0^5^	
35-44	6.86^**^	1.91-24.59			8.51^**^	2.33-31.10	9.41^***^	2.57-34.49
45-54	4.79^*^	1.37-16.76			6.18^**^	1.72-22.24	7.22^**^	2.00-26.06
55-64	5.11^**^	1.51-17.27			6.99^**^	2.00-24.40	8.45^***^	2.41-29.62
65-84	4.44^*^	1.28-15.38			6.67^**^	1.85-24.05	3.97^*^	1.10-14.34
Years since last hospitalization	1.13^**^	1.04-1.22			1.19^***^	1.09-1.30	1.19^***^	1.09-1.30
Years with hospitalizations	1.05	0.92-1.21			1.11	0.89-1.38	1.11	0.89-1.38
Number of Hospitalizations	1.01	0.99-1.04			1.01	0.97-1.04	1.01	0.97-1.04

*p < .05 **p < .01 ***p < .001.

^1^First bivariate relationships for the variables hospitalization, survey, gender and age groups were estimated in the entire sample, then bivariate relationships were estimated separately for the hospitalized including the three additional variables, i.e., number of years since hospitalization, number of hospitalizations during a year, and number of years with hospitalizations.

^2^In Models 1 and 2 the variables hospitalization, survey, gender, and age group were included in the first model for the entire sample, while the second model estimated relationships among previously hospitalized. Besides gender and age group, the second model for the previously hospitalized also included the three variables: number of years since the last hospitalization, the number of hospitalizations during a year, and the number of years with hospitalizations.

^3^In Model 3 contrasts among those with previous hospitalizations show the interaction terms, i.e., whether the OR between surveys among the hospitalized differ significantly from the corresponding OR for the non-hospitalized. In this case OR was 1.37 times larger than 0.69.

^4^The contrast shows the interaction term, i.e., OR was 0.76 times that of 1.85.

^5^The contrasts show the interaction terms. Since age group 23–34 is the reference category its deviation corresponds to OR between the hospitalized and the not hospitalized in general. Thus, for age group 23–34 OR for the hospitalized was 0.11 times that of the non-hospitalized (because the corresponding OR for the non-hospitalized in this age group is 1.0).

**Table 4:** **Odds ratios (OR) for non-hazardous alcohol use from logistic regression models with previous alcohol-related hospitalization, gender, age group, and interactions between hospitalization and; gender, age group, numbers of years after hospitalization, number of hospitalizations during a year, and number of years with hospitalizations as independent variables**

	Bivariate ^1^		Model 1 ^2^		Model 2		Model 3	
	OR	95 CI	OR	95 CI	OR	95 CI	OR	95 CI
**All**								
Non-hospitalized	1.0		1.0				1.0	
Hospitalized	0.38^***^	0.32-0.45	0.38^***^	0.32-0.45			0.42^*^	0.21-0.84
2006	1.0		1.0				1.0	
2002/2007 survey	2.30^***^	2.20-2.39	2.32^***^	2.23-2.42			2.34^***^	2.25-2.44
Men	1.0		1.0				1.0	
Women	0.86^***^	0.83-0.90	0.84^***^	0.81-0.87			0.84^***^	0.81-0.88
Age group								
23-34	1.0		1.0				1.0	
35-44	1.29^***^	1.21-1.37	1.23^***^	1.16-1.31			1.24^***^	1.16-1.32
45-54	1.04	0.97-1.10	0.98	0.92-1.04			0.98	0.92-1.04
55-64	1.09^**^	1.02-1.16	1.01	0.95-1.08			1.02	0.95-1.08
65-84	1.04	0.98-1.10	0.94	0.89-1.00			0.94^*^	0.88-0.99
**With previous alcohol-related hospitalizations**								
2006	1.0				1.0		1.0^3^	
2002/2007 survey	1.23	0.87-1.73			1.23	0.86-1.76	0.53^***^	0.37-0.75
Men	1.0				1.0		1.0^4^	
Women	0.79	0.56-1.11			0.82	0.58-1.17	0.98	0.68-1.40
Age group								
23-34	1.0				1.0		1.0^5^	
35-44	0.68	0.36-1.31			0.71	0.36-1.37	0.57	0.29-1.11
45-54	0.74	0.41-1.34			0.77	0.42-1.42	0.78	0.43-1.45
55-64	0.99	0.57-1.71			1.04	0.58-1.84	1.02	0.57-1.82
65-84	1.68	0.94-3.00			1.63	0.89-3.00	1.75	0.95-3.22
Years since last hospitalization	1.01	0.96-1.07			1.01	0.96-1.09	1.02	0.96-1.09
Years with hospitalizations	0.92	0.83-1.03			1.21	0.96-1.52	1.21	0.96-1.52
Number of Hospitalizations	0.96^*^	0.94-0.99			0.92^*^	0.87-0.98	0.92^*^	0.87-0.98

*p < .05 **p < .01 ***p < .001.

^1^First bivariate relationships for the variables hospitalization, survey, gender and age groups were estimated in the entire sample, then bivariate relationships were estimated separately for the hospitalized including the three additional variables, i.e., number of years since hospitalization, number of hospitalizations during a year, and number of years with hospitalizations.

^2^In Models 1 and 2 the variables hospitalization, survey, gender, and age group were included in the first model for the entire sample, while the second model estimated relationships among previously hospitalized. Besides gender and age group, the second model for the previously hospitalized also included the three variables: number of years since the last hospitalization, the number of hospitalizations during a year, and the number of years with hospitalizations.

^3^In Model 3 contrasts among those with previous hospitalizations show the interaction terms, i.e., whether the OR between surveys among the hospitalized differ significantly from the corresponding OR for the non-hospitalized. In this case OR was 1.37 times larger than 0.69.

^4^The contrast shows the interaction term, i.e., OR was 0.76 times that of 1.85.

^5^The contrasts show the interaction terms. Since age group 23–34 is the reference category its deviation corresponds to OR between the hospitalized and the not hospitalized in general. Thus, for age group 23–34 OR for the hospitalized was 0.11 times that of the non-hospitalized (because the corresponding OR for the non-hospitalized in this age group is 1.0).

**Table 5:** **Odds ratios (OR) for hazardous alcohol use from logistic regression models with previous alcohol-related hospitalization, gender, age group, and interactions between hospitalization and; gender, age group, numbers of years after hospitalization, number of hospitalizations during a year, and number of years with hospitalizations as independent variables**

	Bivariate ^1^		Model 1 ^2^		Model 2		Model 3	
	OR	95 CI	OR	95 CI	OR	95 CI	OR	95 CI
**All**								
Non-hospitalized	1.0		1.0				1.0	
Hospitalized	2.58^***^	2.16-3.08	2.41^***^	2.01-2.89			6.12^***^	2.97-12.62
2006	1.0		1.0				1.0	
2002/2007 survey	0.36^***^	0.34-0.38	0.36^***^	0.34-0.38			0.35^***^	0.34-0.37
Men	1.0		1.0				1.0	
Women	0.82^***^	0.78-0.86	0.83^***^	0.79-0.87			0.83^***^	0.79-0.87
Age group								
23-34	1.0		1.0				1.0	
35-44	0.75^***^	0.70-0.81	0.78^***^	0.73-0.85			0.79^***^	0.73-0.85
45-54	1.07	0.99-1.15	1.13^**^	1.05-1.21			1.13^**^	1.05-1.22
55-64	1.02	0.96-1.10	1.09^*^	1.01-1.17			1.10^**^	1.03-1.19
65-84	0.62^***^	0.58-0.67	0.67^***^	0.62-0.72			0.68^***^	0.63-0.73
**With previous alcohol-related hospitalizations**								
2006	1.0				1.0		1.0^3^	
2002/2007 survey	0.79	0.55-1.15			0.84	0.57-1.23	2.36^***^	1.59-3.49
Men	1.0				1.0		1.0^4^	
Women	1.13	0.79-1.62			1.01	0.69-1.48	1.22	0.83-1.80
Age group								
23-34	1.0				1.0		1.0^5^	
35-44	0.68	0.35-1.32			0.60	0.30-1.18	0.76	0.38-1.50
45-54	0.79	0.44-1.44			0.64	0.34-1.19	0.56	0.30-1.06
55-64	0.55^*^	0.32-0.97			0.44^**^	0.24-0.80	0.40^**^	0.22-0.73
65-84	0.31^***^	0.16-0.58			0.25^***^	0.13-0.49	0.37^***^	0.19-0.72
Years since last hospitalization	0.91^**^	0.85-0.97			0.88^***^	0.82-0.94	0.88^***6^	0.82-0.94
Years with hospitalizations	1.06	0.94-1.18			0.88	0.72-1.08	0.88	0.72-1.08
Number of Hospitalizations	1.02	1.00-1.04			1.04	0.99-1.08	1.04	0.99-1.08

*p < .05 **p < .01 ***p < .001.

^1^First bivariate relationships for the variables hospitalization, survey, gender and age groups were estimated in the entire sample, then bivariate relationships were estimated separately for the hospitalized including the three additional variables, i.e., number of years since hospitalization, number of hospitalizations during a year, and number of years with hospitalizations.

^2^In Models 1 and 2 the variables hospitalization, survey, gender, and age group were included in the first model for the entire sample, while the second model estimated relationships among previously hospitalized. Besides gender and age group, the second model for the previously hospitalized also included the three variables: number of years since the last hospitalization, the number of hospitalizations during a year, and the number of years with hospitalizations.

^3^In Model 3 contrasts among those with previous hospitalizations show the interaction terms, i.e., whether the OR between surveys among the hospitalized differ significantly from the corresponding OR for the non-hospitalized. In this case OR was 1.37 times larger than 0.69.

^4^The contrast shows the interaction term, i.e., OR was 0.76 times that of 1.85.

^5^The contrasts show the interaction terms. Since age group 23–34 is the reference category its deviation corresponds to OR between the hospitalized and the not hospitalized in general. Thus, for age group 23–34 OR for the hospitalized was 0.11 times that of the non-hospitalized (because the corresponding OR for the non-hospitalized in this age group is 1.0).

^6^The estimate is pooled; the 2006 OR was 0.96, the 2002/2007 OR was 0.72, and the difference was significant (p < .001).

### Abstinence

The bivariate associations are expressed as relative odds in Table 3. They indicate that the prevalence of abstinence was higher among hospitalized than among non-hospitalized respondents, lower in 2007 than in 2006, and higher for women than for men. Abstinence rates were also generally higher for the youngest age group in comparison with the older age groups. The one exception was the oldest age group (65–84), which had the highest prevalence.

Among the previously hospitalized, the odds for abstinence did not differ significantly between the two surveys or by gender. In comparison with the youngest age group (23–34), the odds of abstinence were higher in all the other older age groups. Odds for abstinence also increased significantly with number of years since last hospitalization.

Models 1 and 2 showed that including all variables in the same models attenuated the estimates somewhat, but the overall pattern in the results remained the same.

Model 3 showed the results corresponding to models 1 and 2 when variables from both the models were included in the same model, but with alternative contrasts for the hospitalized. The first block of rows in Table 3 corresponds to the relative odds among the non-hospitalized. That is, in comparison with Model 1, the estimated odds ratios can now be said to exclude the hospitalized. In comparison with Model 2, the last rows for the hospitalized show contrasts reflecting the interaction terms. Relative odds are given for the non-hospitalized in comparison with the hospitalized. That is, the estimated odds ratios show whether the odds ratios for the hospitalized deviate significantly from the odds ratios for the non-hospitalized for the different categories of the independent variables. The interaction terms in Model 3 indicate that the odds ratios for abstinence did not differ significantly for the hospitalized in comparison with the non-hospitalized for the survey and gender variables, but they were significantly different among the hospitalized with regard to age group.

Models 2 and 3 also showed that the odds for abstinence increased with number of years since last hospitalization, even when adjusted for survey, gender, age group, years with hospitalization, and number of hospitalizations.

### Non-hazardous use

The bivariate associations in Table 4 indicate that the odds for non-hazardous use were lower among hospitalized than among non-hospitalized respondents, higher in the 2007 study than in the 2006 study, and lower for women than for men. The odds for non-hazardous use were similar across the different age groups, with the exception of the 35–44 and 55–64 groups, which were associated with somewhat higher prevalence.

Model 1, for the outcome, non-hazardous use, showed that including all variables in the same model attenuated the bivariate estimates for the non-hospitalized, although the overall pattern of results remained the same.

As shown in Table 4, Model 2 indicated that, among the previously hospitalized, the odds for non-hazardous use did not differ significantly between surveys, by gender, or by age group. The odds for non-hazardous use decreased with an increasing number of hospitalizations.

Model 3 showed only one significant interaction term for the hospitalized in comparison with the non-hospitalized. It indicated that the higher estimate for non-hazardous use in the 2002/2007 survey in comparison with the 2006 survey deviated significantly for the hospitalized. The odds ratio of 1.23 between the surveys (from Model 2) among the hospitalized was significantly lower (0.53 times lower) than the corresponding odds ratio of 2.32 (from Model 1) for the non-hospitalized.

### Hazardous use

In Table 5, the bivariate associations, and also Model 1, indicated that the odds for hazardous use were higher among hospitalized than among non-hospitalized respondents, lower in the 2007 study than in the 2006 study, and lower for women than for men. The odds for hazardous use were similar in the youngest age group compared with the older age groups, with the exceptions of the 35–44 and 65–84 age groups, which had somewhat lower prevalence rates.

Among the previously hospitalized, both the bivariate associations and Model 2 indicated that the odds for hazardous use did not differ significantly between surveys or by gender. Older age groups were associated with lower odds for hazardous use, significantly so for the 55–64 and 65–84 age groups. The odds for hazardous use decreased with increasing number of years since last hospitalization. A comparison of the estimates for models 1 and 2 with the bivariate results suggests that, although the inclusion of additional variables in models 1 and 2 attenuated the estimates, the pattern in the results remained similar.

The significant interaction terms for the hospitalized in Model 3 indicated that the survey and age group relationships deviated significantly between the hospitalized and the non-hospitalized. The age group relationships were significantly different, and the OR of 0.84 between the surveys (from Model 2) among the hospitalized was significantly higher (2.36 times larger) than the OR of 0.35 (from Model 3) among the non-hospitalized.

The bivariate odds for hazardous use decreased with number of years since last hospitalization. As models 2 and 3 indicated, this was true even after adjustment for survey, gender, age group, years with hospitalization, and number of hospitalizations.

Further analysis indicated that, while the proportion of hospitalized decreased over time in one sample, there was a non-significant change in the other sample over time. A comparison between the samples indicated that they showed different trends, yet when pooled, the two samples indicated a significant decrease in hazardous use over time following hospitalization.

## Discussion

Our results show that previously hospitalized persons are characterized by a lower probability of non-hazardous use; they are, however, not only more likely to be hazardous users but also more likely to become abstainers as time passes following hospitalization. Evidence of continuing excessive alcohol use has also been indicated previously by their high rate of return to alcohol-related care [6].

Of persons with previous alcohol-related morbidity, one in six reported abstinence, and half non-hazardous use. While non-hazardous use was found to be less prevalent than in the general population, our estimate is considerably higher than in previous studies of other treatment populations [25]. As expected, hazardous use was found to be more prevalent than in the general population. The extent of hazardous use in the general population in this study is in line with previous population estimates [4].

The likelihood of non-hazardous use decreases with number of hospitalizations. This is in line with the general finding that prior hospitalization is one of the strongest predictors of further hospitalization [26].

In line with a previous study, we found the level of hazardous use to be higher for men than for women among people in general [4]. Among the previously hospitalized, there seem to be no significant gender differences in non-hazardous use, abstinence, and hazardous use. This finding contradicts some earlier treatment studies, which suggest that women fair better following treatment [6, 14, 27].

Almost half were found to be hazardous users in the youngest age group among the previously hospitalized. This percentage decreased gradually with age, and one fifth were hazardous users in the oldest age group (65–84). Although abstinence was lower in the youngest age group among the previously hospitalized, there were no significant age differences in non-hazardous use. The greater extent of hazardous use in younger ages appears to contradict the previous finding that they are less likely to return for further alcohol-related hospital care [6, 28]. However, people in the youngest age group are at the beginning of their alcohol-usage history, which may account for their lower probability of receiving further care despite their greater extent of hazardous use.

The proportion of persons who did not change (32 percent with hazardous use at follow-up) lies within the range of estimates indicated by previous studies on other treatment populations [29–32]. However, previous estimates vary widely, probably due to variations in inclusion criteria, study design, and measurement differences – besides divergences in treatment effects. For example, in a larger American population-based study with retrospective data, 28 percent of the treated individuals, compared with 24 percent of those who were never treated, were still dependent on alcohol one year later when assessed using DSM-IV criteria [33]. According to another study, between 17 and 79 percent of the persons who went through treatment had not recovered when assessed at follow-up [29]. In a treatment-outcome study in Stockholm County, 63 percent were still heavy users after one year, while the corresponding estimate in a US study sample was 46 percent [34].

Abstinence has historically been recognized as the successful treatment outcome for people with alcohol-use disorders [25, 35–40], although some high consumers have periods of abstinence followed by relapse. The estimated abstinence rate following treatment ranges from 34 to 64 percent [30, 31, 36, 41]. Some of the persons diagnosed as alcohol dependent develop non-hazardous use following treatment, where estimates have ranged from 3 to 32 percent [25, 41]. In the previously mentioned treatment-outcome study of hospitalized inhabitants of Stockholm, 23 percent reported non-hazardous use, while 14 percent were abstinent one year after treatment [34].

The current results are in line with the suggestion of some treatment-programs, e.g., Alcoholics Anonymous, that abstinence may be necessary to change hazardous habits among some persons [25, 31, 32]. In our study, previously hospitalized persons appeared to drift towards abstinence, whereas the aggregated extent of non-hazardous use after the hospitalization event remained similar over time and lower than among persons who had not been hospitalized. Previous treatment studies have indicated a mixed picture [29–32, 42–50]. Although some data support the notion of an increased abstinence rate over time after treatment [29], no study has formally addressed this particular issue. The closest is a study that has proposed that development over time may be best characterized as an increase in stochastic change in behavior [45]. This is an issue that deserves further study.

It seems reasonable that alcohol-related behaviors associated with this particular at-risk group tend to be more unstable. Repeated changes in alcohol behavior are likely to contribute to lower estimates of hazardous use. Given the high risk of returning to alcohol-related inpatient care, our estimates of hazardous use may actually be quite low.

Registry data in its current form provide valuable information, but such data can be of greater value if additional information is collected. Additional information on alcohol consumption and a classification into types of behavioral treatment provided would be beneficial. Follow-up data would also be informative. For example, records of six-month post-treatment visits, or a telephone or internet survey of alcohol use, could provide important time-based information that would potentially enable monitoring of treatment outcomes and provide information for future reforms (e.g., changes in treatment protocols). Such evaluation should cover all registered cases. More restrictive inclusion criteria are likely to affect success rates negatively. Such follow-up may require increased efforts to reach the targeted population, possibly in mixed modes or even with proxy reports, since previously hospitalized persons are known to be more likely to become non-responders.

Despite rather high recidivism, reflected in a high rate of return to alcohol-related inpatient care, a majority of people hospitalized for alcohol-related issues report non-hazardous alcohol use or abstinence. This information is important to bear in mind when treating hospitalization as a negative outcome of excessive alcohol use. There is a need for future research that enables overtime comparison between people who receive inpatient care and those who receive other forms of treatment or no treatment at all.

### Limitations

In epidemiological monitoring, measures of self-reported alcohol consumption are a necessity. Without such data, it would not be possible to study distributions and trends among subpopulations and risk groups. Nonetheless, it is well-known that all self-reports underestimate consumption in comparison with sales figures. The data-collection mode, e.g., web, mail, telephone, or face-to-face interview, seems to play only a minor role [51, 52]. Still, even if prevalence estimates may be skewed, trends should be unbiased, given that under-reporting probably remains the same over time.

This study was based on a mixed cohort; even among the hospitalized, a substantial proportion were without an alcohol-disorder diagnosis (e.g., harmful use or dependence). Differences in inclusion criteria suggest that our results may not be comparable with those of other studies that use treatment samples. In particular, in the current study, brief therapy and prior assessment by health care personnel may have affected the estimated rates of hazardous use among people without a dependence or harmful-use diagnosis.

Another limitation of this study is that it is unclear what hospitalization involved in terms of treatment. Although the effects of counseling are well understood, not all patients will have chosen to receive such treatment. Studies have indicated that even being questioned at an ordinary health care reception may be related to decreased alcohol use [53]. Earlier research has indicated that outpatient care following hospitalization decreases the risk of re-hospitalization [14]. Our results suggest that it may be important to perform follow-ups with patients over a longer time period to monitor changes in alcohol use and treatment effects.

While it may be presumed that the hospitalized had hazardous use at the time of their hospitalization, this may not necessarily have been the case. The current study was not able to control for this possibility because there was no measurement of their alcohol use at this time. Also, the measurement instruments used may have been poor at capturing the alcohol habits of this particular population. The instruments require that respondents assess their average habit, something that may be difficult for persons whose consumption varies considerably. The PSNW measure asks for the consumed volume of alcohol a typical week, and AUDIT for the average daily, weekly, or monthly number of glasses during the preceding year.

The group of hazardous users in the longitudinal study sample delimited by AUDIT-C was smaller, suggesting stricter selection of participants. However, this might be deceptive because the lower rate of hazardous use may also have been caused by the selective attrition associated with longitudinal studies.

The sampling frames for both studies were similar, but one was cross-sectional, whereas the other was a follow-up to a baseline study. The follow-up study had attrition, so its non-response rate was higher than in the cross-sectional study. Potential bias associated with the higher non-response rate for the follow-up study may have affected our results. It is well-known that attrition, as well as initial non-response, is related to alcohol use. At the same time, it seems less plausible that attrition explains the finding of decreased hazardous use over time in just one of the two samples.

In general, non-response together with selective mortality is likely to skew results towards healthier behaviors. A previous study has shown that persons who have been hospitalized for alcohol-related reasons are more than two times more likely to become non-responders [54]. Future analyses of selection effects could be made, possibly by linking registry data in longitudinal designs, with repeated-measurements samples, including data on persons who die or move between baseline and follow-up.

There is also a need to validate registry data concerning the alcohol-related diagnoses. For example, there may be underreporting due to the social stigma associated with these diagnoses.

Another study limitation is inherent in its design. It is a limitation shared with most longitudinal studies. In our case, the influences due to the passage of time since last hospitalization were potentially confounded by the changes in health care between 1996 and 2007. This also means that there are competing interpretations of the results. The decreasing abstinence rate may have been the result of a gradually diminishing treatment effect during the follow-up period. Alternatively, it may have come about through changes in the selection of people who received treatment during the follow-up period; that is, the characteristics of patients changed in such a way that abstinence after treatment decreased continuously during the decade under study. It should be noted that the number of persons who underwent alcohol-related hospitalization during any one year increased in Stockholm between 1997 and 2007 [5]. Such interpretations, however, seem implausible to us, but they cannot be dismissed on grounds of the present data.

In sum, differences in measures and design between the surveys, random variability between years, and selective mortality may all have affected the results in general and the differences in results between the two samples. Overall, a decrease in hazardous use was observed over time.

Our investigation focused on alcohol-related hospitalization. Its findings should not be generalized to minor and more common consequences of hazardous drinking.

## Conclusions

Half the group with previous morbidity reported non-hazardous alcohol use. Still, hazardous use was found to be more prevalent among persons with previous morbidity, particularly in the youngest age group. In the years following alcohol-related hospitalization, people seem to drift towards sobriety. Although the changes in hazardous use are inconclusive, the pooled results indicate a decrease in hazardous use over the time following an episode of alcohol-related hospitalization. This result implies that extended follow-up may be needed adequately to capture changes in alcohol use after treatment. Targeted interventions, such as individual health care surveillance, focusing, for example, on abstention over a longer period of time among younger persons who have received alcohol-related hospital care, may prove beneficial.
